# Editorial: Endocrine-related cardiovascular diseases: Recent advances in diagnosis and treatment

**DOI:** 10.3389/fendo.2023.1147752

**Published:** 2023-01-31

**Authors:** Audesh Bhat, Arti Dhar, Kaushik Desai

**Affiliations:** ^1^ Center for Molecular Biology, Central University of Jammu, Jammu and Kashmir, India; ^2^ Department of Pharmacy, Birla Institute of Technology and Sciences Pilani, Telangana, India; ^3^ Department of Anatomy, Physiology & Pharmacology, University of Saskatchewan, Saskatoon, SK, Canada

**Keywords:** cardiovascular disease, lipocalin-2 (LCN-2), Alpha-adrenergic receptor, diffuse myocardial fibrosis, calMR, Chronic coronary syndrome (CCS), thyroid hormones and carotid plaque, essential hypertension (HTN)

Cardiovascular diseases (CVDs) are a group of diverse pathological conditions of the heart and the vascular system with different etiology. CVDs are the number one cause of human mortality and per the WHO’s latest estimates, 32% of global mortality is due to cardiovascular-related diseases (WHO, 2022). The cardiovascular system (CVS) is greatly influenced by our endocrine system in multiple ways, ranging from the regulation of blood pressure through an intricate network of hormonal signaling involving the Renin-Angiotensin system (RAS) to the positive or negative chronotropic effect (1). Several decades of painstaking research have helped us to understand these regulations in detail to a large extent with more and more facets being discovered every year. These efforts have helped in designing new diagnostic and therapeutic approaches to tackle life-threatening cardiovascular complications.

This editorial summarizes the contribution to the special issue of Frontiers of Endocrinology “*Endocrine-related Cardiovascular Diseases: Recent Advances in Diagnosis and Treatment*”. This issue was launched with the aim to provide a platform for the research community working in the areas of CVDs to share their findings on our recent understanding of the role of the endocrine system in CVDs, its diagnosis, and treatment. A total of five articles; four original and one systematic review were selected for publication among the numerous submissions received. A brief summary of each contribution is highlighted below.

Diabetes often leads to secondary complications, impacting almost all vital organs. The impact of diabetes on kidney and heart function is well documented. In the first article that was accepted for publication, Gan et al. explored the relationship between lipocalin-2 (LCN-2), an acute phase protein released by neutrophils in response to multiple stresses (2) and carotid atherosclerotic plaque (CAP) and diabetic nephropathy (DN) in 749 type II diabetic patients. Their study revealed a significant association between the high serum levels of LCN-2 and CAP or DN which increased further in DN with CAP group. Besides CAP and DN, the authors also reported a positive correlation between high levels of LCN-2 and many other pathophysiological factors, such as hypertension, uric acid, and creatinine levels, decreased estimated glomerular filtration rate, and urinary albumin-to-creatinine ratio. Although known to have a potential role in the formation of atherosclerosis (3), this study further adds to our knowledge that perhaps the stress-induced release of LCN-2 has multiple pathological effects on the functioning of our vital organs, including the cardiovascular system and kidneys.

The adrenal glands, especially the medulla, is an important mediator of endocrine regulation of blood pressure and cardiovascular function through circulating epinephrine and norepinephrine. The relationship between mental stress and blood pressure is well established. However, the mechanisms of adrenergic receptors, especially alpha receptors, in hyperreactivity to circulating catecholamines in essential hypertension are not known. In the second article of this collection entitled “Alpha-adrenergic mechanisms in the cardiovascular hyperreactivity to norepinephrine-infusion in essential hypertension”, Walther et al. described the role of alpha-adrenergic receptor (α-AR) in essential hypertension. The authors show that there is selective dysfunction of alpha 1 receptors in mediating systolic blood pressure hyperreactivity to norepinephrine infusion, whereas these receptors mediate diastolic blood pressure hyperreactivity in essential hypertensive individuals, which can be inhibited by a non-selective alpha receptor blocker. Thus, either the alpha receptors do not mediate systolic hyperreactivity or else their function is impaired, which sets the stage for investigating the underlying molecular mechanisms and its possible implications for appropriate therapeutic approaches.

Diffuse myocardial fibrosis (DMF) poses an increased health risk of progressing towards heart failure due to several adverse changes that occur in the heart, such as diastolic dysfunction, increased myocardial stiffness, and left ventricular remodeling (4). If stopped from progressing through timely intervention, DMF can be reversed. In their article entitled “Empagliflozin reduces diffuse myocardial fibrosis by extracellular volume mapping: A meta-analysis of clinical studies” Wang et al. performed a meta-analysis on the effect of empagliflozin on DMF. Empagliflozin belongs to the growing list of antidiabetic drugs that inhibit sodium-glucose cotransporters (SGLTs) but have also been shown to possess cardiovascular benefits, including a reduction in DMF (5). Out of the 244 articles that were identified by searching different databases and by using relevant keywords, a total of six studies were found to meet the inclusion criteria. The authors concluded from their analysis that empagliflozin offers benefits to patients suffering from DMF and can be used as an effective strategy to reverse DMF.

The development of easy, fast, cost-effective, reliable, and preferable non-invasive methods is the ultimate aim of modern disease detection approaches. It becomes more imperative when detecting life-threatening diseases, including cardiovascular diseases. Over several decades of technological innovation, many techniques have been developed to assess cardiac function. The use of invasive coronary flow rate (CFR) and microcirculatory resistance (IMR) techniques to assess coronary microvascular function is quite prevalent with each technique having its own merits and demerits, especially when assessing coronary microvascular function in obese patients (6). In the fourth article entitled “Prognostic impact of coronary microvascular dysfunction assessed by caIMR in overweight with chronic coronary syndrome patients”, Feng et al. demonstrated the use of the coronary angiography-derived index of microcirculatory resistance (calMR), a less-invasive and pressure-wire free index to assess the coronary microvascular function in the chronic coronary syndrome (CCS) patients with obesity. The authors found that calMR value of >=25 was independently associated with major adverse cardiac events in obese patients but not in non-obese patients. Their data supports a significantly higher prognostic value of calMR in obese patients suffering from CCS, thus a potential biomarker of CCS in obese patients.

In the fifth article entitled “Impaired sensitivity to thyroid hormones and carotid plaque in patients with coronary heart disease: A RCSCD-TCM study in China” Liu et al analyze the association between thyroid hormone sensitivity and risk of carotid plaque in patients with coronary heart disease (CHD) and further explore this association based on baseline characteristics and lifestyle. This was a retrospective cross-sectional study and included 6679 CHD patients (aged 35-75). Central sensitivity to thyroid hormone was evaluated by the thyroid feedback quantile-based index (TFQI), thyroid-stimulating hormone index (TSHI), and thyrotroph thyroxine resistance index (TT4RI). Peripheral sensitivity to thyroid hormone was assessed by the free triiodothyronine/free thyroxine (FT3/FT4) ratio. Out of 6679 CHD patients, 72.50% had a carotid plaque. In the multi-adjusted models, the TFQI, TSHI, and TT4RI were positively associated with the risk of carotid plaque. Compared with females and people > 60 years, the odds ratio (OR) value for the carotid plaque was higher in males and people ≤ 60 years. smokers and drinkers had higher OR values for carotid plaque than non-smokers and non-drinkers. FT3/FT4 ratio was negatively associated with carotid plaque, and the OR value for the carotid plaque was lower in males, patients ≤ 60 years, smokers, and drinkers. In conclusion, this study showed that thyroid hormone sensitivity is associated with carotid plaque in patients with CHD. This association is more significant in male smokers and drinkers aged ≤ 60 years.

## Author contributions

AB was the guest associate editor and wrote the paper text. AD was the guest associate editor of the Research Topic and edited the text. KD was the guest associate editor of the Research Topic and edited the text. All authors contributed to the article and approved the submitted version
